# Allergic anaphylaxis due to subcutaneously injected heparin

**DOI:** 10.1186/1710-1492-9-1

**Published:** 2013-01-10

**Authors:** Diana Anders, Axel Trautmann

**Affiliations:** 1 Department of Dermatology, Venereology, and Allergology, University Hospital of Würzburg, Josef-Schneider-Str. 2, Würzburg, 97080, Germany

**Keywords:** Anaphylaxis, Allergy, Basophil activation test, Enoxaparin, Heparin, Hypersensitivity, Immunoglobulin E, Immediate-type

## Abstract

Heparins are one of the most used class of anticoagulants in daily clinical practice. Despite their widespread application immune-mediated hypersensitivity reactions to heparins are rare. Among these, the delayed-type reactions to s.c. injected heparins are well-known usually presenting as circumscribed eczematous plaques at the injection sites. In contrast, potentially life-threatening systemic immediate-type anaphylactic reactions to heparins are extremely rare. Recently, some cases of non-allergic anaphylaxis could be attributed to undesirable heparin contaminants.

A 43-year-old patient developed severe anaphylaxis symptoms within 5–10 minutes after s.c. injection of enoxaparin. Titrated skin prick testing with wheal and flare responses up to an enoxaparin dilution of 1:10.000 indicated a probable allergic mechanism of the enoxaparin-induced anaphylaxis. The basophil activation test as an additional *in-vitro* test method was negative. Furthermore, skin prick testing showed rather broad cross-reactivity among different heparin preparations tested.

In the presented case, history, symptoms, and results of skin testing strongly suggested an IgE-mediated allergic hypersensitivity against different heparins. Therefore, as safe alternative anticoagulants the patient could receive beneath coumarins the hirudins or direct thrombin inhibitors. Because these compounds have a completely different molecular structure compared with the heparin-polysaccharides.

## Background

Heparins and heparinoids are widely used for prevention and treatment of thromboembolic diseases or during haemodialysis. Unfractionated heparin (UFH), low-molecular-weight heparins (LMWHs), fondaparinux, and the heparinoid danaparoid are indirect anticoagulants which require the cofactors thrombin, antithrombin, and factor Xa to exert their activity. UFH is a heterogenous mixture of glycosaminoglycans (anionic polysaccharides) that bind to antithrombin via a unique pentasaccharide sequence and catalyse the inactivation of thrombin, factor Xa and other clotting enzymes. LMWHs are derived from UFH by chemical or enzymatic depolymerisation. They comprise about one third the molecular weight of UFH with a mean of 4.000 to 5.000 kilo Dalton (kDa). Compared with UFH, LMWHs have a greater inhibitory activity against factor Xa, have superior pharmacokinetic properties, a longer half-life, and are associated with a significant lower risk of heparin-induced thrombocytopenia (HIT). Fondaparinux, a synthetic pentasaccharide, specifically inhibits factor Xa in an antithrombin-dependent fashion. For patients with HIT, three parenteral direct thrombin inhibitors and danaparoid are currently approved as alternatives to heparin [1].

Considering the widespread use of heparins in daily medical in- and outpatient practice hypersensitivity reactions to heparins are rare. The delayed-type hypersensitivity (DTH) reactions to s.c. injected heparin preparations are well-known, usually presenting as itchy erythematous or eczematous plaques directly around the injection sites. In spite of some research in this area, the decisive antigenic determinants of the heparin molecule have not yet been identified. A rather characteristic feature of heparin-DTH is the extensive cross-reactivity among UFH, all available LMWHs, and danaparoid. Fondaparinux is also an anionic polysaccharide and cross-reactivity is likely, especially if prolonged treatment periods are necessary [2]. Theoretically, heparin-DTH implies the risk of a generalised exanthema or eczema if heparin is administered i.v. Importantly, patients with DTH to s.c. injected heparins usually tolerate heparin i.v., a phenomenon referred to as compartment allergy [3].

In contrast to DTH, immediate-type anaphylactic reactions after heparin administration are extremely rare. In 2008, several cases of immediate-type reactions after i.v. injected UFH could be clarified as non-allergic anaphylaxis due to contaminants [4]. In the patient presented herein, an IgE-mediated allergy to heparins is suggested by convincing history and skin testing results.

## Case presentation

A 43-year-old male patient presented to our allergy clinic after a severe anaphylactic episode for further allergologic work-up. He had simply forgotten oral anticoagulation with phenprocoumon and thus alternatively injected 80 mg enoxaparin s.c. himself at home into the skin of the abdomen. 5–10 minutes later he was seriously affected by nausea, sweating, dizziness, erythema, and intense generalised itching. Blood pressure decreased to 70/40 mm Hg as documented by the emergency physician. The patient first showed tachycardia with frequencies up to 140 beats per minute, then he developed an intermittent junctional escape rhythm with frequencies of 40 beats per minute. He heavily vomited. Within 15 minutes he was treated by the emergency physician i.v. with 250 mg prednisolone, 5 mg clemastine, 200 mg cimetidine and 0.5 mg epinephrine. Following emergency treatment he entirely recovered and the cardiopulmonary surveillance did not show any more abnormalities. On further questioning his wife affirmed that there was an impressive wheal directly at the injection site of enoxaparin at the abdomen in size of the palm of a hand whereas she described the rest of the skin as blazing red. Outwards oral anticoagulation with phenprocoumon was restarted without any overlapping heparin application. Afterwards he reported that he has also eaten some peanuts just before the anaphylaxis episode.

The personal history comprised a factor V Leiden mutation in a heterozygous form. Recurrently, he had experienced thromboembolic events like pulmonary emboli and deep venous thromboses. For this reason and due to additional cardiovascular risk factors like obesity, hypercholesterolemia, hyperhomocysteinemia and diabetes mellitus he previously was advised to continuous oral anticoagulation with phenprocoumon. Enoxaparin was applied during total endoprosthesis of the right and left hip joint 5 and 3 years ago.

Laboratory investigations including serum IgE level, baseline serum tryptase and IgE to whole peanut extract and the recombinant peanut allergens rAra h 1, 2, 3, 8 and 9 showed no pathological findings. Histamine-controlled skin prick testing with native peanuts performed on the volar forearm remained negative. Thereafter, our patient still consumed peanuts finally ruling out IgE-mediated peanut allergy as cause of anaphylaxis.

Skin prick testing with the UFH heparin-sodium, LMWHs (nadroparin, dalteparin, enoxaparin), heparinoids (danaparoid, pentosan polysulfate) and fondaparinux with reading at 20 minutes revealed the strongest positive reaction to enoxaparin and additional positive reactions to heparin-sodium, nadroparin, danaparoid and fondaparinux (Table 1). We additionally performed a dilution prick test series with enoxaparin including 1:10, 1:100, 1:1.000, 1:10.000 and 1:100.000 dilutions (Table 2). Even with the 1:10.000 dilution of enoxaparin, corresponding to 100 ppm, a positive skin test reaction was provoked. Therefore, an IgE-mediated hypersensitivity to enoxaparin was strongly suggested. A basophil activation test (BAT) with heparin-sodium, pentosan polysulfate, danaparoid, fondaparinux, dalteparin, enoxaparin and nadroparin failed to reveal any positive results.

**Table 1:** Skin prick testing with UFH, LMWHs, heparinoids and fondaparinux

**Drug tested**	**Wheal diameter [mm]**
Heparin-sodium 25.000 I.E./ml	3
Nadroparin (Fraxiparin™) 9.500 I.E./ml	4
Dalteparin (Fragmin™) 12.500 I.E./ml	0
Enoxaparin (Clexane™) 10.000 I.E./ml	6 and pseudopodes
Danaparoid (Orgaran™) 1.250 I.E./ml	3
Pentosan polysulfate (Fibrezym™) 100 mg/ml	0
Fondaparinux (Arixtra™) 5 mg/ml	4
Sodium chloride 0.9% solution	0
Histamine 10 mg/ml	5

Reading at 20 minutes. Each test was performed with the undiluted heparin preparations, i.e. the pure therapeutic heparin solution.

**Table 2:** Skin prick testing dilution series with enoxaparin 10.000 I.E./ml in sodium chloride 0.9% solution

**Drug tested**	**Wheal diameter [mm]**
Enoxaparin (Clexane™) 1:10 (10%)	6
Enoxaparin (Clexane™) 1:100 (1%)	5
Enoxaparin (Clexane™) 1:1.000 (0,1%)	5
Enoxaparin (Clexane™) 1:10.000 (100 ppm)	3
Enoxaparin (Clexane™) 1:100.000 (10 ppm)	0
Sodium chloride 0.9% solution	0
Histamine 10 mg/ml	7

Reading at 20 minutes. ppm, parts per million.

## Conclusions

Up to now, immediate-type hypersensitivity reactions to heparins were published very rarely. In the literature we found 9 documented cases of immediate-type hypersensitivity reactions to heparins (Table 3). Unfortunately, a sufficient allergological work-up suggesting IgE-mediated allergic hypersensitivity was performed only in 5 of these cases.

**Table 3:** Published cases of immediate-type hypersensitivity reactions to heparins

**No.**	**Age (years)**	**Sex**	**Causative heparin**	**Clinical symptoms**	**Skin test (causative heparin)**	**BAT (causative heparin)**	**Ref.**
1	36	m	Porcine heparin	Blood pressure decrease, airway stoppage, heart fibrillation	SPT (titrated) positive	n.d.	[9]
2	30	f	Dalteparin	Urticaria, nausea, dyspnea, swollen hands	SPT positive IDT (1:100) positive	n.d.	[6]
3	52	m	Heparin	Hypotension, tachycardia, loss of consciousness	SPT negative IDT (undiluted) positive	n.d.	[8]
4	42	f	Nadroparin	Generalised urticaria, angioedema, hypotension, collapse	SPT positive	n.d.	[7]
5	18	f	Enoxaparin	Erythematous infiltrated plaques, angioedema	SPT negative, IDT (diluted?) positive	positive	[5]
6	27	f	Enoxaparin	Erythematous infiltrated plaques, angioedema, dizziness, sweating	SPT negative, IDT negative	negative	[5]
7	59	f	Dalteparin	Blood pressure decrease, frequent arrhythmias, dyspnea	n.d.	negative	[11]
8	52	f	Reviparin	Dyspnea, cough, wheezing	n.d.	n.d.	[12]
9	67	f	Heparin	Cardiac and respiratory arrest	n.d.	n.d.	[13]

f = female; m = male; SPT = skin prick test; IDT = intradermal test; n.d. = not done.

The allergological work-up of immediate-type hypersensitivity reactions to heparins rely on skin prick and intradermal heparin testing with readings after 15 to 20 minutes. Specificity of heparin skin testing seems to be high if clearly non-irritating heparin concentrations were used whereas the sensitivity of this testing procedure is largely unknown. Generally, lower heparin concentrations (i.e. higher dilutions) increase the specificity but may decrease the sensitivity of testing. Therefore, as a first screening concentration for heparin hypersensitivity it is recommended to use the undiluted therapeutic heparin solutions for prick testing and a 1:10 dilution for intradermal testing, respectively [2].

Using this approach, i.e. undiluted therapeutic heparin concentrations for prick testing and a 1:10 dilution for intradermal testing, immediate-type test reactions may be observed in up to 10% of cases. These reactions should not be naively interpreted as proof of an IgE-mediated allergy because they could be caused by an unspecific heparin-induced histamine liberation. These falsely positive reactions have to be discriminated from extremely rare immediate-type allergic reactions by more extensive skin testing using a further series of dilutions (1:100, 1:1.000, 1:10.000). In true allergic reactions these lower concentrations should still yield positive results.

Accordingly, the presented patient demonstrated positive prick test results up to a 1:10.000 dilution of enoxaparin suggesting IgE-mediated allergy. The enoxaparin preparation used for skin testing was composed of enoxaparin and aqua without further additives. Further, the observed cross-reactivity throughout a panel of heparin preparations from different manufacturers tested ruled out a causal role of contaminants or preservatives added to some products, such as sodium metabisulfite, benzyl alcohol, or chlorocresol. BAT was proposed as a complementary method for *in-vitro* diagnosis of heparin allergy [5]. But until now, the results of these authors could not be confirmed by further published data. Moreover, we and other groups with experience in the field of heparin allergy repeatedly failed to detect heparin sensitization by BAT.

Harr et al. diagnosed IgE-mediated allergy to s.c. injected dalteparin by positive skin prick and intradermal tests in a patient with generalised urticaria accompanied by nausea and mild dyspnea. Surprisingly, skin test-negative UFH was tolerated in an i.v. challenge test [6]. Van Zuuren reported of a patient with local urticarial reactions at the injection sites of nadroparin, once followed by generalised urticaria, angioedema and collapse. They reported cross-reactivity with skin-test negative enoxaparin because in a subcutaneous challenge test generalised urticaria developed [7]. Berkun and colleagues referred to a patient with heparin-induced recurrent anaphylaxis during haemodialysis with hypotension and loss of consciousness confirmed by positive intradermal skin testing with UFH and LMWHs. One hour after a heparin-induced anaphylactic episode an elevated serum tryptase level was measured by enzyme-linked immunosorbent assay which returned to normal within 24 hours [8]. Merely historically, allergic anaphylaxis to heparin preparations were attributed to protein contaminants of animal origin during suboptimal production processes, namely with porcine gut-derived heparin preparations [9].

Non-allergic heparin-associated anaphylaxis may be caused by direct histamine release from mast cells and basophils by nonspecific binding of contaminants or indirectly by complement/kinin activation. In 2008, oversulfated chondroitin sulfate and dermatan sulfate could be proven as contaminants of heparin in a series of patients with anaphylactic reactions. Oversulfated chondroitin sulfate activates the kallikrein-kinin system with generation of bradykinin and activation of the potent anaphylatoxins C3a and C5a both leading to anaphylaxis symptoms [4].

In case of therapeutic necessity for immediate anticoagulation in our patient strict avoidance of all heparins is mandatory. Alternatively, he could receive hirudins or direct thrombin inhibitors both exhibiting a complete different molecular structure compared with the heparin-polysaccharides. Argatroban as a competitive inhibitor of thrombin is a small molecule with a molecular weight of 500 kDa. It is administered as continuous i.v. infusion resulting in a plasma half-life of 45 minutes, which could be monitored by the thromboplastin time. It is licensed for treatment and prevention of thrombosis associated to HIT and for anticoagulation during percutaneous coronary interventions when heparin is contraindicated [1]. Dabigatran is an orally taken direct thrombin inhibitor approved for prophylaxis of deep vein thrombosis after total hip and total knee arthroplasty. Recently, the approval was expanded for the prevention of stroke in patients with atrial fibrillation [10].

Here we reported an extremely rare case of heparin-induced anaphylaxis assured by skin prick testing up to impressively very high heparin dilutions. Additionally, rather broad cross-reactivity between available polysaccharide-anticoagulants, such as UFH, LMWHs and heparinoids, was observed. In such a situation beneath hirudins the recently approved direct thrombin inhibitors argatroban or dagibatran are potential alternatives because of their completely different chemical structure.

This publication was funded by the German Research Foundation (DFG) and the University of Wuerzburg in the funding program Open Access Publishing.

## Consent

Written informed consent was obtained from the patient for publication of this case report and any accompanying images. A copy of the written consent is available for review by the Editor-in-Chief of this journal.

## Abbreviations

(UFH): Unfractionated heparin; (LMWHs): Low-molecular-weight heparins; (DTH): Delayed-type hypersensitivity; (mm Hg): Millimetre of mercury; (BAT): Basophil activation test; (ppm): Parts per million; (kDa): Kilo Dalton.

## Competing interest

The authors declare that they have no competing interests.

## Authors’ contributions

AT designed the allergological work-up and supervised the interpretation of the data. DA performed the allergological work-up, composed and finalised the manuscript. Both authors revised and approved the manuscript finally.
